# Impact of corn processing and weaning age on calf performance, blood metabolites, rumen fermentation, and behavior

**DOI:** 10.1038/s41598-023-37728-3

**Published:** 2023-07-03

**Authors:** A. Rahimi, G. R. Ghorbani, F. Hashemzadeh, M. Mirzaei, H. R. Saberipour, F. Ahmadi, M. H. Ghaffari

**Affiliations:** 1 Department of Animal Sciences, College of Agriculture, Isfahan University of Technology, Isfahan, 84156–83111 Iran; 2 Department of Animal Science, Faculty of Agriculture and Natural Resources, Arak University, Arak, 38156-88349 Iran; 3 Department of Animal Sciences, College of Agriculture and Natural Resources, University of Tehran, Tehran, Iran; 4 School of Agriculture, Food and Ecosystem Sciences, Faculty of Science, The University of Melbourne, Parkville, VIC 3010 Australia; 5 Institute of Animal Science, University of Bonn, 53115 Bonn, Germany

**Keywords:** Developmental biology, Physiology

## Abstract

This study examined the impact of two corn processing methods (steam-flaked (SFC) vs. ground) combined with two weaning ages (50 or 75 days) on calf performance, blood metabolites, rumen fermentation, nutrient digestion, and behavior. The study involved 48 three-day-old Holstein calves, with an average body weight of 41.4 ± 2.2 kg. The experimental design was a 2 × 2 factorial arrangement, resulting in four treatment groups: SFC50 (SFC and weaning at 50 days), SFC75 (SFC and weaning at 75 days), GC50 (ground corn and weaning at 50 days), and GC75 (ground corn and weaning at 75 days). Calves were given whole milk at 4 L/ day from day 3–15 and 7 L/ day from day 16 to either 43 or 68, depending on weaning age. Weaning occurred between days 44 and 50 for early-weaned calves and between days 69 and 75 for late-weaned calves. The study lasted until calves were 93 days old. The starter ration consisted of soybean meal, corn grain, 5% chopped wheat straw and premix. Results indicated that the SFC-based starter feed improved calf performance and nutrient digestion, as evidenced by increased weight gain, dry matter, crude protein, and neutral detergent fiber digestibility. Calves fed the SFC-based starter diet had lower blood albumin and urea N concentrations, while blood total protein and globulin concentrations were higher, especially in early-weaned calves. No significant changes in rumen pH and ammonia-N concentration were observed. In addition, the SFC starter feed resulted in higher volatile fatty acids concentration and longer feeding time in weaned calves compared to ground corn. Overall, these results suggest that an SFC-based starter feed may be beneficial for both early and late weaned calves.

## Introduction

Promoting the intake of solid feed before weaning can lead to faster rumen development and improved nutrient digestion, which is critical for optimal calf growth and development^1^. The incorporation of processed cereal grains into starter formulations for young calves has been shown to improve nutrient digestion^2,3^, promotes functional rumen fermentation^4,5^, improves weight gain^3,6^, and increases feed efficiency^7,8^.

According to Offner et al. ^9^, steam-flaked grains showed the highest digestibility, followed by fine grinding and dry rolling. In steam flaking, the semi-crystalline starch in the grains is gelatinized at high moisture temperature and pressure, which facilitates microbial binding in the rumen and increases starch digestion in ruminants ^10^. This is particularly important for young calves, as increased production of propionate and butyrate due to increased starch digestion can stimulate the development of rumen papillae^11,12^. Bateman et al.^13^ reported that feeding starters high in fines reduced calf feed intake and growth, but calf performance did not differ between starters containing steam-flaked or dry-rolled corn grain. Similarly, Ghassemi-Nejad et al.^14^ found that calf growth improved when a starter diet containing steam-flaked grains was fed compared to a starter diet in ground form. On the other hand, feeding calves a starter feed with a high fines content has been shown to lower rumen pH, inhibit growth and activity of cellulolytic bacteria, and possibly impair rumen health and function ^15^. The addition of forage has been shown to stabilize rumen fermentation when processed grains are included in the initial calf diet ^16,17^.

Milk quantity and age at weaning can affect physiological and morphological rumen development in calves. Incomplete rumen development can prevent calves from efficiently utilizing nutrients in starter feed after weaning, resulting in poor growth performance and increased susceptibility to disease^18,19^. High liquid feed intake during the milk-feeding phase can reduce nutrient digestion after weaning^20^. Prolonging the milk feeding strategy by postponing weaning to an age greater than 8 weeks has been shown to mitigate the decline in body weight associated with weaning^21,22^. However, a late weaning program can slow calf rumen development, reducing solid feed intake and nutrient digestion capacity and hindering full growth potential^23^. Conversely, limited milk supply in the preweaning period can negatively affect calf behavior and growth performance^24^. Early weaning programs or milk restriction during the milk feeding period have been proposed as management strategies to improve the profitability of calf rearing programs^25^. Lopreiato et al.^26^ found that early weaning (45 vs. 60 days) had no significant effect on behavior and rumen development in post-weaned calves, resulting in a reduction in calf rearing costs. Similarly, Bjorklund et al.^25^ reported that despite lower weight gains and body measurements in calves weaned at 30 days compared to calves weaned at 60 or 90 days, all calves eventually reached similar final weights at 120 days of age. Jasper and Weary^27^ and Khan et al.^28^ found that feeding large amounts of milk during the preweaning period resulted in increased growth rate before weaning and increased body weight after weaning. However, Hill et al.^29^ observed poor feeding efficiency during and after weaning. In addition, feeding large amounts of milk may discourage calves from eating starter feeds, which delays rumen development and functionality and possibly reduces nutrient digestion^27,30^.

There is a paucity of information on the interaction between grain processing methods and weaning age on calf performance. Our hypothesis is that increased digestibility of steam-flaked corn (SFC) will result in higher metabolizable energy intake from solid feed for earlier weaned calves (50 vs. 75 days), offsetting lower energy intake from liquid feed and promoting the development of functional rumen fermentation, leading to improved growth performance. The objective of this study is to evaluate the effects of corn processing methods (steam-flaked or ground) and weaning age (50 or 75 days) on calf performance, blood metabolites, rumen fermentation, nutrient digestion, and behavior.

## Materials and methods

The experiment was conducted from January to May 2022 on a commercial dairy farm located in Avin Dasht, Ghazvin, Iran. Ethical approval for all procedures involving animals was obtained from the Animal Care and Use Committee of Isfahan University of Technology (IUT, Iran; IACUC #2021/C02) before the start of the study. All methods were performed following the Animal Care and Use Committee of the Iranian Council for Animal Care. The study complies with ARRIVE guidelines for reporting in vivo experiments and all methods were performed in accordance with the relevant guidelines and regulations. Weather data, including average ambient temperatures (°C; T) and relative humidity (%; RH), were obtained from a meteorological station near the farm (approximately 1000 m from the farm). Temperature-humidity index (THI) was calculated according to the formula of Vitali et al.^31^: THI = 0.8 × average T + (average RH /100) × (maximum T—14.4) + 46.4. Throughout the experiment, the average daily values for temperature, relative humidity, and THI were 11.0 ± 6.04, 49.8 ± 15.96, and 53.0 ± 7.80, respectively.

### Calf management

Newborn calves were weighed and housed in individual straw-bedded pens (1.2 × 1.8 m) shortly after birth. The bedding was replaced daily to maintain cleanliness and dryness. The first two feedings consisted of 2.5 L of high-quality colostrum (Brix value > 22) delivered via nipple bottle. The quality of the colostrum was determined using a PAL-1 Brix refractometer (Atago Co. Ltd., Bellevue, WA, USA), and it was discarded if the Brix value was below 22^32^. The first portion of colostrum was fed within 30–60 min of birth, and the second portion was fed approximately 12 h later. Blood was collected from the jugular vein 24 h after the first colostrum feeding, and the total serum protein concentration was determined using a portable digital refractometer (VET 360; Reichert Inc., Depew, NY, USA). Only calves with a total serum protein concentration greater than 5.5 g/dL were eligible to participate in the study. From the second colostrum feeding until day 3 of life, transition milk was offered twice daily (0900 and 1700 h), and from day 3, pasteurized whole milk was offered in two equal portions twice daily (0800 and 1600 h).

### Corn grain processing

The same batch of whole corn grain was used for both fine milling and steam flaking. The raw corn grains were finely ground in a hammer mill (model 5543 GEN, Isfahan Dasht, Isfahan, Iran) with a 3-mm sieve, which was used specifically for the preparation of starter feeds that required finely ground corn. Steam flaking was performed in a commercial feed processing facility using a flaker (Lantus C22129091 Chavdaneh Plant, Isfahan, Iran). The method used was based on the approach described by Plascencia and Zinn^33^ with modifications previously reported by Mojahedi et al.^16^. To briefly summarize the procedure, whole corn grains were steamed for 30 min at a constant temperature of 99 °C and a pressure of 5.44 atm. This process took place in a vertically oriented steam chamber before the rolling process was initiated. Subsequently, the steamed corn grains were passed through corrugated rollers with a size of 46 × 90 cm, resulting in the production of corn flakes with a density of about 0.39 kg/L. Determination of flake density (measured in kg/L) was performed on freshly processed corn samples collected directly under the roller. The processed flakes were then allowed to cool on a concrete pad. Both steam-flaked and ground corn were mixed directly with other starter feed ingredients such as soybean meal, straw, and premix without undergoing a pelleting process. The steam-flaked corn retained its flaked structure and contributing to the texture of the final feed. As for the physical form of the starters, the characteristic structures of the steam-flaked corn were retained in the final mixture, resulting in a textured feed.

### Experimental design, and treatments description

**Figure 1:**
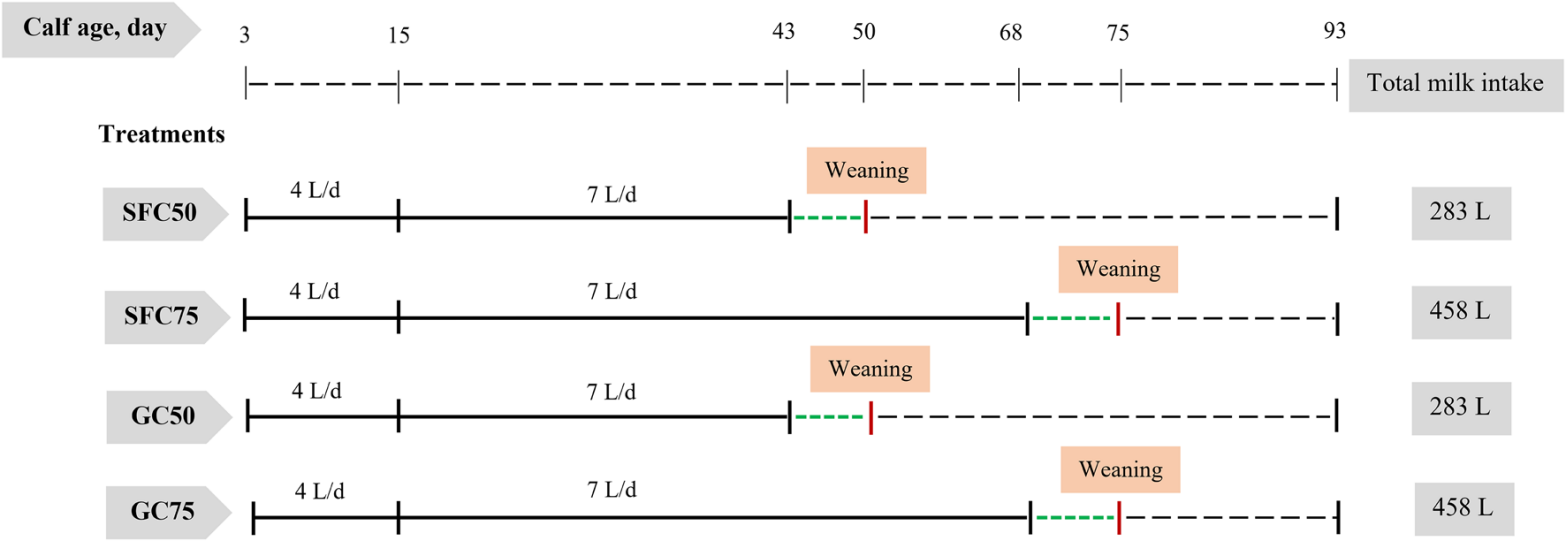
Schematic diagram represents a 2 × 2 factorial arrangement of treatments, resulting in 4 treatments. (1) calves fed starter diet containing steam-flaked corn (SFC) and weaned on d 50 of age (SFC50), (2) calves fed starter diet containing SFC and weaned on d 75 of age (SFC75), (3) calves fed starter diet containing ground corn (GC) and weaned on d 50 of age (GC50), and (4) calves fed starter diet containing GC and weaned on d 75 of age (GC75). The experiment was progressed until 90 d of experiment. Calves in SFC50 and GC50 groups received 4 L/d of milk from d 3 to 15 and 7 L/d of milk from d 11 to 43. Weaning occurred from d 44 to 50, with a 0.5-L daily reduction in milk quantity in each meal. Calves in SFC75 and GC75 groups received 4 L/d of milk from d 3 to 15 and 7 L/d of milk from d 11 to 68. Weaning occurred from d 69 to 75, with a 0.5-L daily reduction in milk quantity in each meal.

On day 3 of life, 48 calves (body weight = 41.4 ± 2.20 kg; mean ± SE; 12 calves (6 male and 6 female) per treatment) were randomly assigned to four treatments in a 2 × 2 factorial design (Fig. 1). The experimental factors were (1) corn processing method (steam flaking vs. fine grinding) and (2) age at weaning (50 vs. 75 days). Treatments were as follows: Calves offered a starter ration with steam-flaked corn (SFC) and weaned on day 50 (SFC50) Calves offered a starter ration with SFC and weaned on day 75 (SFC75) Calves offered a starter ration with ground corn (GC) and weaned on day 50 (GC50) Calves offered a starter ration with GC and weaned on day 75 (GC75). Experimental starter diets containing 5% chopped wheat straw were formulated according to the Cornell Net Carbohydrate and Protein System (CNCPS, version 5.1; Table 1). Corn grain, in the form of steam-flaked or ground corn, were the only grain source in the calf starters and were included in the 50.4% dry matter (Table 1). The starters contained similar ingredients but different physical forms of corn grain (steam-flaked vs. finely ground). Calves in the early weaning phase (SFC50 and GC50) received 4 L/day of milk from day 3–15 and 7 L/day of milk from day 16–43. Weaning was gradual from day 44–50, with milk volume reduced by 0.5 L/day at each meal. Calves in the late weaning phase (SFC75 and GC75) received 4 L/day of milk from day 3–15 and 7 L/day of milk from day 16–68. Weaning was gradual from day 69–75, with milk volume reduced by 0.5 L/day at each meal. Milk refusals were negligible and not recorded. All calves remained in the experiment until day 90. Clean water and starter feed were freely available throughout the study. Starter feed was adjusted daily to achieve 5–10% orts, which was collected and weighed daily before morning feeding.

**Table 1:** Ingredients, chemical composition, and particle size distribution of starter ration containing corn grain in steam-flaked or ground form.

Items	Corn processing method	
	Steam-flaking	Fine grinding
Ingredients, % of DM		
Wheat straw, chopped	5.0	5.0
Corn grain, ground	–	50.42
Corn grain, steam-flaked	50.42	–
Soybean meal, 44% crude protein	35.08	35.08
Calcium carbonate	1.67	1.67
Dicalcium phosphate	0.54	0.54
Sodium bicarbonate	1.67	1.67
Magnesium oxide	0.55	0.55
Vitamin and mineral mix^a^	4.19	4.19
Salt	0.89	0.89
Chemical composition, % of DM		
DM	90.0	90.0
Crude protein	21.2	21.0
Ether extract	3.5	3.5
NDF	14.5	14.2
NFC^b^	50.3	50.4
Metabolizable energy, Mcal/kg^3^	2.74	2.71
Calcium^c^	0.96	0.96
Phosphorus^c^	0.51	0.51
Particle size distribution, % retained on sieve (mean ± SD)		
4.75 mm	20.4 ± 1.5	19.6 ± 0.6
2.36 mm	70.4 ± 1.9	25.2 ± 3.0
1.18 mm	3.6 ± 0.3	13.0 ± 0.8
0.6 mm	2.4 ± 0.3	24.6 ± 0.9
0.3 mm	2.0 ± 0.2	13.6 ± 2.4
0.15 mm	0.6 ± 0.3	3.3 ± 0.8
Pan	0.5 ± 0.0	0.6 ± 0.2
Geometric mean particle size^d^, mm	2.40 ± 0.01	1.24 ± 0.09

^a^Vitamin and mineral mix (per kg) contained: vitamin A = 750,000 IU, vitamin D_3_ = 25,000 IU, vitamin E = 500 IU, Ca = 100 g, *P* = 10 g, Mg = 20 g, Zn = 1,000 mg, Cu = 250 mg, I = 50 mg, Co = 60 mg, Mn = 500 mg, Se = 50 mg.

^b^Nonfiber carbohydrates (NFC) = 100 − (NDF + crude protein + ether extract + ash) (NRC, 2001).

^c^Estimated using the NRC (2001) models.

^d^Geometric mean particle size calculated as described by the American Society of Agricultural Engineers (ASAE, 1983).

### Sample collection and analysis

Milk samples were collected biweekly and stored at 4 °C with potassium dichromate for analysis of lactose, protein, fat, and total solids using MilkoScan (Foss Electric, Hillerød, Denmark) according to the methodology described by AOAC International^34^. The starter intake of individual calves was recorded daily, with representative samples of starter feed and orts collected three times per month and stored frozen (− 20 °C) for later analysis. Feces were collected from individual calves for five consecutive days, from day 85–89 of the experiment, to determine the apparent digestibility of the entire tract using the acid-insoluble ash method^35^. The feed, ort, and fecal samples were oven dried (65 °C for 48 h), ground through a 1-mm sieve in a Wiley mill (Ogaw Seiki Co. Ltd., Tokyo, Japan), and analyzed for dry matter (DM, method 930.15), ash (method 942.05), and crude protein (method 990.03) according to the procedures of AOAC International^34^. NDF analysis included α-amylase and sodium sulfite^36^.

Total starch concentration in ground or steam-flacked corn grains were determined using the modified glucoamylase method described by Zhu et al. ^37^. Briefly, samples were gelatinized by boiling in a water bath for 20 min and then cooled to room temperature. After addition of acetate buffer, the starch was hydrolyzed with a glucoamylase enzyme, and the glucose concentration was measured by the glucose oxidase method (Pars Azmoon Co., Tehran, Iran). The same method was used to measure the degree of gelatinization of corn starch, omitting the boiling step. The total starch content of the corn grain was 68.2% (DM). The degree of gelatinization of the starch was 48.8% and 14.1% of the total starch for steam-flacked and ground corn grains, respectively.

Calves were weighed at birth, on day 3 of life, and then every 10 days until the end of the experiment using a calibrated digital scale (model EES-500, Ettehad Inc., Isfahan, Iran). Feed efficiency was determined as body weight gain (kg) per total DMI (milk DM + starter feed DM)^38^. Measurements of skeletal growth (withers height, body length, hip height, heart girth, and hip width) were taken every 10 days until the end of the experiment^39^.

On day 80 of the study, 4 h after morning feeding, rumen fluid was collected via a stomach tube. Rumen pH was determined using a digital pH meter (HI 8318, Hanna Instruments, Cluj-Napoca, Romania), and a sample of rumen fluid was acidified with 25% metaphosphoric acid and stored frozen (− 20 °C) for VFA analysis using a gas chromatograph (model CP -9002, Chrompack, Middelburg, The Netherlands) equipped with a 50-m silica-fused column (CP -Wax Chrompack Capillary Column; Varian Medical Systems, Palo Alto, California, USA), as described by Bal et al.^40^.

On days 40, 60, and 80 of the study, blood samples were collected from the jugular vein in K2 EDTA-containing evacuated tubes (Becton Dickinson Vacutainer Systems, Franklin Lakes, NJ, USA). Samples were immediately centrifuged at 3000×*g* for 15 min at 4 °C, and plasma samples were stored at − 20 °C. Albumin, total protein, urea, glucose, alanine aminotransferase, and aspartate aminotransferase were analyzed using commercial kits according to the manufacturer's instructions (Pars Azmoon Co., Tehran, Iran), and globulin was calculated from the difference of total protein and albumin. β-hydroxybutyric acid (BHB) levels were quantified with an autoanalyzer using a commercial kit (Randox Laboratories Ltd., Ardmore, UK).

Throughout the experiment, the farm veterinarian monitored the health status of calves daily. Health scores were assessed based on a standardized scoring system (CalfTrack) developed by Lesmeister and Heinrichs ^41^. The consistency of the feces was rated on a 5-point scale, with 1 representing normal and 5 representing watery consistency, mucus, and bloody^41^. The general appearance of the calves was also rated on a 5-point scale, with 1 representing normal and alert and 5 representing severe lethargic^41^. Calf respiration was scored on a 5-point scale, with 1 representing normal and 5 representing severe and chronic cough^41^. The number of days with a fecal score ≥ 3 was used as an indicator of abnormal fecal consistency. Abnormal general appearance or respiration was recorded if the value was ≥ 3. The rectal temperature of calves was determined daily at approximately 14:00 h using a veterinary thermometer (Yasa Teb Co., Isfahan, Iran) inserted into the rectum for 1 min. Rectal temperature was also categorized according to the number of days with a temperature ≥ 39.4 °C, which corresponds to the fever threshold^42^. Each calf diagnosed with the disease received veterinary treatment according to standard farm procedures.

Behavioral activities of the calves were observed for three consecutive days (78–80 of the experiment) from 08:00 to 16:00 during daylight hours. Two trained individuals, unaware of the treatments, recorded calf behavior. A rumination period was defined as at least one observation of rumination activity that occurred after at least 5 min of no rumination^43^.

### Statistical analysis

A standard deviation of 100 g average daily gain (ADG) was assumed, with the minimum meaningful difference in ADG set at 65–75 g/day, based on the values published previously^44^. A power test analysis was run (α = 0.05 and power (1–β) = 0.80), and the expected sample size of 12 calves/treatment was achieved for growth performance, the most reliable parameter for power test determination. Data were analyzed using a completely randomized design with treatments arranged in a 2 × 2 factorial design (corn processing method (steam-flaking vs. fine grinding) and weaning age (50 vs. 75 d) as the main factors), using the MIXED procedure of SAS (SAS 9.4, SAS Institute Inc., Cary, NC). All residuals were tested for normality using the Shapiro–Wilk statistic and the UNIVARIATE procedure of SAS as well as for homogeneity of variance using Levene's test and visually assessed using quantile–quantile plots. Data were log-transformed (base 10) if the assumptions of normality of residuals were not met. The data repeated over time were analyzed including time as repeated measures. Individual calf was the experimental unit. The experimental factors (corn processing, weaning age, and their interaction), time (d) and calf sex were the fixed effects in the model. Calf within treatment was a random factor. Toeplitz, autoregressive type 1, and compound symmetry were tested and the covariance structure was selected if it minimized Schwarz's Bayesian information criterion. The initial values of body measures were used as covariate for the corresponding variables. Contrast statements were constructed to distinguish differences in the main experimental factors and their interactions. Using the GLIMMIX procedure of SAS with a Poisson distribution and a log link function, the treatment effect on categorical responses (health scores) was examined including time as repeated measures. The threshold of significance was set at *P* ≤ 0.05 and trends were discussed at 0.05 < *P* ≤ 0.10. The number of days calves were treated and the number of days with abnormal feces, appearance, or respiratory score (≥ 3) were analyzed using the GENMOD procedure of SAS with a Poisson distribution.

## Results

**Figure 2:**
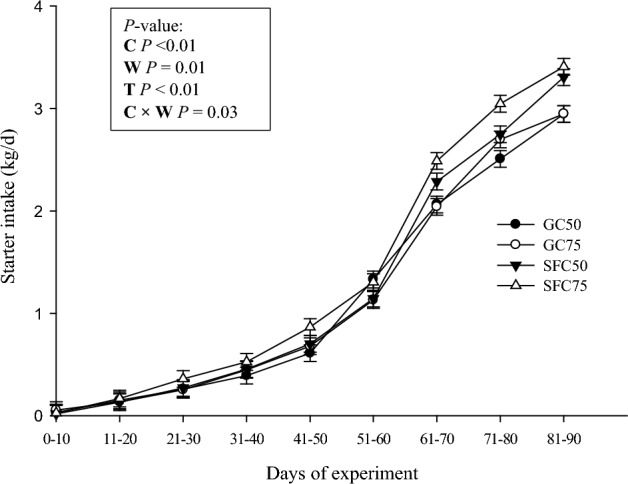
Calf starter intake (least square mean ± SEM) in 10-d intervals from d 1 to 90 of experiment in calves fed starter diet containing steam-flaked corn (SFC) and weaned on d 50 of age (SFC50), 2) calves fed starter diet containing SFC and weaned on d 75 of age (SFC75), 3) calves fed starter diet containing ground corn (GC) and weaned on d 50 of age (GC50), and 4) calves fed starter diet containing GC and weaned on d 75 of age (GC75). C = corn processing method. W = weaning age. T = measurement time.

**Figure 3:**
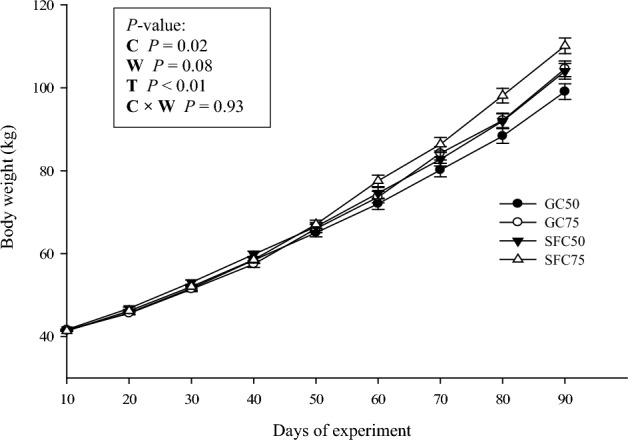
Body weight (least square mean ± SE) in calves fed starter diet containing steam-flaked corn (SFC) and weaned on d 50 of age (SFC50), 2) calves fed starter diet containing SFC and weaned on d 75 of age (SFC75), 3) calves fed starter diet containing ground corn (GC) and weaned on d 50 of age (GC50), and 4) calves fed starter diet containing GC and weaned on d 75 of age (GC75). C = corn processing method. W = weaning age. T = measurement time.

**Figure 4:**
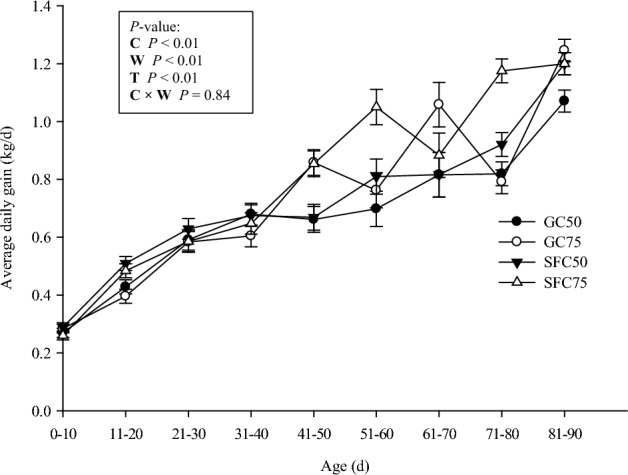
Average dairy gain (least square mean ± SE) in calves fed starter diet containing steam-flaked corn (SFC) and weaned on d 50 of age (SFC50), 2) calves fed starter diet containing SFC and weaned on d 75 of age (SFC75), 3) calves fed starter diet containing ground corn (GC) and weaned on d 50 of age (GC50), and 4) calves fed starter diet containing GC and weaned on d 75 of age (GC75). C = corn processing method. W = weaning age. T = measurement time.

The starter feed containing SFC had a coarser particle size distribution and a geometric mean particle size of 2.4 mm, while the GC-containing feed had a finer particle size distribution and a geometric mean particle size of 1.24 mm (Table 1). The interaction effect between the corn processing method and weaning age was significant for starter feed intake (Fig. 2), with the highest value recorded in SFC75 calves (*P* = 0.01). Calves fed SFC showed higher total starter intake and heavier body weight (Fig. 3) compared to GC-fed calves (*P* = 0.02 and *P* = 0.08, respectively). However, there was no interaction effect between corn processing method and weaning age on ADG. The highest and lowest ADG were recorded in SFC75 (0.79 kg/d) and GC50 (0.67 kg/d), respectively (Fig. 4. *P* < 0.01).

Table 2 shows that early-weaned calves had better feed efficiency compared to late-weaned calves, but there was no effect of the corn processing method on total feed efficiency. Calves fed SFC had a smaller heart girth (2.2 cm; *P* = 0.01) but a larger hip width (0.5 cm; *P* = 0.01) compared to GC-fed calves. Regardless of the corn processing method, late-weaned calves had a longer body length (0.9 cm; *P* = 0.02) and a larger heart girth (1.20 cm; *P* = 0.05) than early-weaned calves. There was a significant interaction effect between weaning age and corn processing method on body length and hip width, with the greatest value recorded in SFC75 calves (*P* < 0.01).

**Table 2:** Effects of corn processing method (steam-flaking vs. grinding) in starter diet and weaning age (50 vs. 75) on calf performance and frame size (n = 12 per treatment).

Items	SFC		GC		SEM	Contrast, *P*-value						
	50	75	50	75		C	W	T	C × W	C × T	W × T	C × W × T
Performance												
Total DM intake (d 1–90), kg/d	1.63	1.99	1.54	1.79	0.03	< 0.01	< 0.01	< 0.01	0.03	0.01	< 0.01	0.86
ME intake, Mcal/d (d 1–90)	5.95	7.59	5.68	6.97	0.08	< 0.01	< 0.01	< 0.01	0.03	0.01	< 0.01	0.86
Feed efficiency (d 1–90)^a^	0.51	0.44	0.47	0.44	0.02	0.35	0.01	< 0.01	0.20	< 0.01	0.01	0.01
Feed efficiency/ME	0.130	0.105	0.119	0.105	0.01	0.24	< 0.01	< 0.01	0.23	< 0.01	< 0.01	0.01
Frame dimensions^b^												
Body length, cm	48.2	50.0	48.8	48.8	0.39	0.43	0.02	< 0.01	0.02	0.04	< 0.01	0.01
Heart girth, cm	92.1	93.9	94.9	95.5	0.63	0.01	0.05	< 0.01	0.39	0.31	< 0.01	0.58
Body barrel, cm	103	101	102	99.3	0.97	0.36	0.01	< 0.01	0.45	0.78	0.01	0.52
Hip height, cm	87.9	88.8	88.8	87.7	0.40	0.81	0.87	< 0.01	0.01	0.02	0.01	0.01
Hip width, cm	17.3	17.6	17.1	16.8	0.14	0.01	0.92	< 0.01	0.04	0.01	0.53	0.23
Withers height, cm	86.8	87.0	87.9	87.0	0.36	0.15	0.28	< 0.01	0.11	0.05	0.92	0.01

*SFC* steam-flaked corn, *GC* ground corn

*C* corn processing method, *W* weaning age. *T*= measurement time.

Total DM intake = milk, starter feed plus wheat straw.

*ME* metabolizable energy.

^a^Feed efficiency = average daily gain/total DM intake^38^.

^b^Frame size measures were determined in 10-d intervals from d 1 to 90 of the experiment.

Table 3 showed that calves fed GC had higher urea-N concentration (*P* < 0.01) and albumin concentration (*P* = 0.04) compared to SFC-fed calves. Late-weaned calves had higher glucose concentration (90.2 vs. 96.9 mg/dL; *P* = 0.02) but lower total protein concentration (5.76 vs. 5.55 g/dL; *P* = 0.01) than early-weaned calves. The interaction effect between corn processing method and weaning age tended to be significant for glucose concentration (*P* = 0.07), with the highest concentration found in GC75 calves.

**Table 3:** Effects of corn processing method (steam-flaking vs. grinding) in starter diet and weaning age (50 vs. 75) on blood variables (n = 12 per treatment).

Items	SFC		GC		SEM	Contrast, *P*-value						
	50	75	50	75		C	W	T	C × W	C × T	W × T	C × W × T
Glucose, mg/dL	92.5	93.9	87.9	99.8	2.99	0.81	0.02	< 0.01	0.07	0.97	0.27	0.02
Blood urea nitrogen, mg/dL	5.65	5.45	5.79	5.81	0.19	< 0.01	0.77	< 0.01	0.13	0.51	0.51	0.51
Total protein, g/dL	5.72	5.48	5.79	5.61	0.09	0.24	0.01	0.02	0.68	0.09	0.43	0.84
Albumin, g/dL	3.18	3.12	3.23	3.23	0.04	0.04	0.46	< 0.01	0.49	0.82	0.42	0.15
Globulin, g/dL	2.54	2.36	2.55	2.38	2.39	0.82	0.06	0.99	0.93	0.15	0.29	0.43
Albumin: globulin	1.32	1.36	1.29	1.38	1.38	0.88	0.34	0.11	0.75	0.68	0.21	0.54
BHB, mmol/L	0.20	0.21	0.22	0.19	0.02	0.95	0.61	< 0.01	0.28	0.58	0.68	0.22
Aspartate aminotransferase, IU/L	49.2	56.7	51.1	52.7	3.03	0.74	0.12	0.83	0.32	0.62	0.31	0.78
Alanine aminotransferase, IU/L	14.1	16.3	15.8	15.6	0.81	0.52	0.24	0.01	0.13	0.37	0.24	0.46

*SFC* steam-flaked corn, *GC* ground corn.

*C* corn processing method, *W* weaning age, *T* measurement time.

*BHB*** = **β-hydroxybutyric acid.

Table 4 showed that weaning age and corn processing method had no effect on rumen pH and ammonia-N concentration, which averaged 5.70 and 6.61 mg/dL, respectively. Calves fed SFC had higher total VFA concentration (160 vs. 132 mM; *P* = 0.02) compared to GC-fed calves. The molar proportions of individual VFA were unaffected by weaning age, with the exception of valerate, which was present in a higher proportion in early-weaned calves. Feeding a starter diet containing SFC compared to GC resulted in a lower molar proportion of butyrate and isovalerate but a higher molar proportion of propionate (*P* = 0.06).

**Table 4:** Effects of corn processing method (steam-flaking vs. grinding) in starter diet and weaning age (50 vs. 75) on ruminal fermentation variables determined on d 80 of the experiment (n = 12 per treatment).

Items	SFC		GC		SEM	Contrast, *P*-value		
	50	75	50	75		C	W	C × W
Ruminal pH	5.64	5.54	5.79	5.81	0.19	0.20	0.64	0.57
Ammonia, mg/dL	5.46	5.94	6.61	7.21	0.86	0.17	0.54	0.94
Total VFA, m*M*	167	153	123	140	11.8	0.02	0.88	0.19
Individual VFA, mol/100 mol								
Acetate	52.4	51.8	53.3	50.7	1.28	0.96	0.21	0.42
Propionate	33.3	35.1	30.1	33.2	1.37	0.06	0.08	0.61
Butyrate	8.62	8.53	10.6	10.2	0.75	0.02	0.73	0.81
Iso-butyrate	0.34	0.41	0.57	0.91	0.15	0.22	0.20	0.40
Valerate	4.73	3.21	4.20	3.30	0.58	0.71	0.04	0.79
Isovalerate	0.50	0.87	1.06	1.57	0.30	0.04	0.15	0.80
Acetate: propionate	1.57	1.48	1.82	1.55	0.10	0.15	0.10	0.38

*SFC* steam-flaked corn, *GC* ground corn, *C* corn processing method, *W* weaning age.

The data on nutrient digestion are presented in Table 5. The results show that feeding starter rations containing SFC (8.2%; P < 0.01) resulted in greater digestion of dry matter (DM), organic matter (4.6%; *P* = 0.01), crude protein (5.4%; *P* = 0.01), and NDF (6.0%; *P* = 0.04) compared to feeding GC. The age at weaning did not affect nutrient digestion, except for the digestibility of DM, which was higher in early-weaned calves (4.55%; *P* = 0.01). No significant interaction effect was observed between weaning age and corn processing method on nutrient digestibility.

**Table 5:** Effects of corn processing method (steam-flaking vs. grinding) in starter diet and weaning age (50 vs. 75) on total-tract apparent nutrient digestibility determined from d 85 to 89 of the experiment (n = 12 per treatment).

Items	SFC		GC		SEM	Contrast, *P*-value		
	50	75	50	75		C	W	C × W
Dry matter, %	74.0	70.1	66.5	61.3	1.17	< 0.01	0.01	0.66
Organic matter, %	76.0	75.4	73.2	69.0	1.75	0.01	0.18	0.31
Crude protein, %	71.0	71.1	68.1	63.2	2.18	0.01	0.25	0.25
Neutral detergent fiber, %	50.4	50.2	48.7	39.9	2.88	0.04	0.12	0.13

*SFC* steam-flaked corn, *GC* ground corn, *C* corn processing method. *W* weaning age. C × W = interaction.

The data on calf health status indicators are presented in Table 6. The results indicate that the corn processing method did not affect calf rectal temperature, respiration rate, and general appearance scores. Late-weaned calves had a lower fecal score (*P* = 0.07) and slightly higher rectal temperature (*P* < 0.01) than early-weaned calves. There was no significant effect of the main experimental factors on the number of abnormal fecal, general, or respiratory days (i.e., score ≥ 3). However, there was a significant interaction (*P* < 0.01) and a tendency towards significance (*P* = 0.07) for days with a rectal temperature ≥ 39.4 °C and days with a respiratory score ≥ 3, with the largest values observed at SFC50 and GC75, respectively. The treatments did not affect the number of days that the animals were medicated, with an average of 1.50 days.

**Table 6:** Effects of corn processing method (steam-flaking vs. grinding) in starter diet and weaning age (50 vs. 75) on calf health status (n = 12 per treatment).

Items^a^	SFC		GC		SEM	Contrast, *P*-value						
	50	75	50	75		C	W	T	C × W	C × T	W × T	C × W × T
Fecal score	1.07	1.06	1.06	1.03	0.01	0.01	0.07	< 0.01	0.52	< 0.01	1.00	0.01
Days with fecal score ≥ 3	1.75	1.33	1.33	1.00	0.34	0.28	0.28		0.90			
Respiratory score	1.18	1.14	1.09	1.21	0.02	0.71	0.02	< 0.01	< 0.01	0.36	1.00	0.63
Days with respiratory score ≥ 3	6.16	4.83	2.91	7.83	1.73	0.94	0.30		0.07			
General appearance score	1.21	1.19	1.14	1.24	0.02	0.44	0.02	< 0.01	0.01	0.08	1.00	0.01
Days with general appearance score ≥ 3	6.08	4.58	4.50	7.66	1.73	0.66	0.63		0.18			
Rectal temperature, ^o^C	38.9	38.7	38.8	38.8	0.01	0.79	< 0.01	< 0.01	< 0.01	0.01	0.12	0.01
Days with rectal temperature ≥ 39.4 °C	9.25	4.08	3.41	7.33	1.53	0.40	0.68		0.01			
Days medicated^b^	1.50	1.50	1.41	1.58	0.44	1.00	0.85		0.85			

*SFC* steam-flaked corn, *GC* ground corn, *C* corn processing method, *W* weaning age. C × W = interaction. *P* = measurement time.

^a^Calf feces was scored on a 5-point scale as 1 = normal and 5 = watery, mucous, and bloody. General appearance was scored on a 5-point scale as 1 = normal and alert, and 5 = severely lethargic. Respiratory assessment was undertaken using a 5-point scale as 1 = normal and 5 = severe and chronic cough.

^b^Total days calves received veterinary medications.

The data on behavioral activities during the 8-h observations from days 75–77 of the experiment are presented in Table 7. The results indicate that there was no interaction between corn processing method and weaning age for any of the recorded calf behaviors. Calves fed a starter ration containing SFC compared to GC spent more time eating (*P* < 0.01) and less time resting (*P* = 0.03). Early-weaned calves spent more time eating and less time drinking than late-weaned calves.

**Table 7:** Effects of corn processing method (steam-flaking vs. grinding) in starter diet and weaning age (50 vs. 75) on calf behavioral activities during 8 h of observations recorded after weaning (from d 75 to 77 of the experiment) (n = 12 per treatment).

Items	SFC		GC		SEM	Contrast, *P*-value		
	50	75	50	75		C	W	C × W
Eating, min	150	142	133	117	3.62	< 0.01	0.01	0.21
Drinking, min	7.77	10.41	7.91	9.44	0.65	0.52	0.01	0.39
Nonnutritive oral behavior, min	40.1	39.8	42.7	47.7	4.64	0.25	0.61	0.57
Rumination, min	136	139	140	135	5.80	0.96	0.88	0.55
Resting, min	144	162	155	170	5.93	0.03	0.41	0.39
Standing, min	167	182	156	171	10.5	0.28	0.15	1.00
Laying, min	312	297	323	308	10.6	0.28	0.15	1.00

*SFC* steam-flaked corn, *GC* ground corn.

*C* corn processing method, *W* = weaning age. C × W = interaction.

## Discussion

We are not aware of any study that has investigated the interaction between the processing method of corn grain (steam-flaked vs. ground) in the calf starters and weaning age when calves were fed forage early in life. An early weaning strategy can be challenging due to welfare issues and calf weight loss after weaning. A late weaning strategy can mitigate the negative effects of weaning stress on calf performance^21,22^. However, the higher cost of milk compared to a basal solid diet is an important factor in delayed milk weaning. Data collected in this trial indicate that SFC benefits calf growth and nutrient digestion at both early and late weaning.

### Starter intake and growth

The literature on the relationship between solid feed intake and calf growth performance in response to starter diets containing cereal grains processed by different methods is inconsistent^45^. For example, Pazoki et al.^6^ found that calves fed a starter diet based on SFC and barley grains had better growth rates and rumen development indicators than calves fed a similar diet containing ground grains. Omidi-Mirzaei et al.^46^ also observed that calves fed a starter ration of steam-rolled barley and SFC had higher growth rates than calves fed a similar coarse ground feed in both the pre- and post-weaning phases. However, Mirzaei et al.^5^ reported that there were no differences in starter feed intake, average daily gain, or skeletal measurements between Holstein calves fed a starter diet containing SFC and barley and those fed a similar diet in ground form.

In contrast, Lesmeister and Heinrichs^41^ found that calves fed a starter diet containing SFC consumed less starter feed, had poorer growth performance, and feed efficiency than calves fed a starter diet containing dry-rolled or whole grains. This difference could be due to the lack of forage in the starter feed formulation necessary to maintain normal rumen fermentation functionality when starch sources with variable fermentability are included in the starter ration for calves^47^. The inclusion of forage in the starter ration for calves stabilizes rumen fermentation and functionality^47^.

Other factors that may affect calf performance include type of feed, physiochemical properties of starter feed^1^, particle size of starter feed^47^, level of milk feeding^48^, and housing system (group or individual)^49^. A decrease in feed intake has been observed when calves are offered a starter feed with a high percentage of fines compared to a feed with larger particles^2,13^. Bateman et al.^13^ reported that total starter feed intake decreased by approximately 0.14 kg/d when the mean particle length of the starter feed decreased from 2.03 to 0.81 mm. The smaller mean geometric particle size of GC-containing starters may explain the lower total starter intake of calves offered GC-containing starters compared to those offered SFC.

Regarding weaning age, Eckert et al.^22^ reported that weaning age had no effect on starter feed intake when calves were offered GC-based starters. However, late-weaned calves consumed more SFC-containing starter feed and showed higher BW and ADG. This increased intake was maintained after weaning as calves consumed more solid feed in the preweaning period, which promoted gastrointestinal and metabolic maturation as indicated by increased rumen VFA concentration, blood BHB concentration, and lower fecal starch concentration^22^. Quigley et al.^19^ attributed the faster growth rate of late-weaned calves to increased intake of solid feed from the liquid feed, which is more digestible and metabolizable than starter feed. The observed differences between the early (d 50) and late (d 75) weaning phases can be explained by changes in physiological age of calves and the amount and duration of milk feeding during the weaning phase^50^.

### Blood metabolites

The increased blood glucose concentration in calves weaned at 75 days compared with calves weaned at 50 days may be due to a higher intake of lactose from milk, which has shown to increase blood glucose levels^51^. The higher blood glucose concentration in late-weaned calves could result in a sparing use of amino acids (AA), leading to their use for growth rather than for the gluconeogenesis pathway^52^. This suggests that late weaned calves had a higher availability of AA to support their increased growth compared to early weaned calves. The results of the current study showed no effect of starter feed containing SFC compared to GC on blood glucose concentration. This was contrary to the expectation that higher digestibility of organic matter might increase blood glucose levels^3^. In addition, there was no difference in blood glucose concentration before and after weaning in calves fed starter diets containing SFC compared to GC^16^. Increasing BHB concentration with calf age reflects increased solid feed intake and is indicative of increased ketogenic activity of the rumen epithelium^53^. The current results differ from the findings of Makizadeh et al.^3^, who reported that calves fed a starter diet containing SFC compared with GC had higher BHB concentrations before weaning (at day 39), but the difference was not observed until after weaning (at day 78).

Neither weaning age nor corn processing method affected blood concentrations of alanine aminotransferase and aspartate aminotransferase, suggesting that they have no effect on healthy calf liver function. The results of the current experiment showed that delayed weaning at 50–75 days of age resulted in lower serum protein concentrations. This observation is consistent with the results of McCoard et al.^54^, who found lower total serum protein concentrations at day 84, but not day 49, in calves weaned later (56 vs. 91 days), which was associated with lower blood urea N and BHB concentrations, suggesting greater rumen wall metabolic activity. However, in the current study, weaning age had no effect on blood urea-N and BHB concentrations, negating a relationship between serum total protein levels and rumen development in calves. Earlier weaning of calves was associated with higher blood globulin concentrations than in later weaned calves, possibly indicating a different pattern in acquisition and/or maintenance of immune components.

### Ruminal fermentation and nutrient digestion

Grinding grains into fine particles can lead to rapid acid production in the rumen and negatively affect the development of the rumen epithelium^55^. This in turn could affect feed intake^56^. However, in the present experiment, rumen pH did not differ between treatments, probably due to the inclusion of chopped wheat straw in the starter diet, which prevented a decrease in rumen pH. Mirzaei et al.^5^ reported that rumen pH of calves offered a starter feed containing corn and barley grains in steam-flaked form without corn silage was 5.25, but increased to 5.89 when the starter feed was mixed with corn silage (15%). This trend was also observed when a starter feed containing corn and barley grains was fed in ground form with or without forage supplementation. Providing effective dietary fiber from forage may help stabilize the rumen environment^16^, and it has been suggested that at least 5% forage should be included in starter feed for calves to prevent a drop in rumen pH^57^. There is also a positive correlation between rumen pH and rumination time, which stimulates salivary secretion and rumen buffering^58^. The lack of differences in rumination time between treatments (Table 7) may also explain why rumen pH was similar among treatments. Organic matter digestibility, determined from day 85–89 of the experiment, was greater in the SFC-based starter compared to the GC-based starter, which likely contributed to the greater VFA concentration on day 80 in the former group due to the greater supply of fermentable organic matter^59^. The greater VFA concentration in calves fed SFC may indicate greater rumen development and microbial fermentation in the rumen. The increase in VFA concentration with calf age could be due to higher intake of solid feed^60^.

The type of processed grain in the starter ration for calves may affect nutrient digestion^2^. van Niekerk et al.^60^ found greater DM, fat and crude protein digestibility and lower NDF digestibility in calves fed SFC compared to whole corn grain in the starter ration. Mojahedi et al.^16^ reported that digestibility values were greater for DM and NDF and lower for crude protein when calves were fed a starter diet containing SFC compared to cracked corn grain. The presence of a large amount of fines in the starter feed for calves reduces the retention time in the rumen, resulting in faster passage of nutrients and lower nutrient digestibility^2,61^. This could explain the lower nutrient digestion in calves offered GC-containing starters. Quigley et al.^62^ found that in addition to the physical form of the grain in the starter feed, calf age, intakes of milk or milk replacer during the preweaning period, and the type and content of carbohydrates in the calf starter formulation are important factors affecting the availability of energy from the calf starter.

Starch digestibility is generally higher for steam-flaked grains than for ground or dry-rolled grains^9^. This is due to the gelatinization of starch in the grains by steam flaking, which increases the accessibility of starch for microbial binding in the rumen^10,63^. The increased availability of energy contributes to more efficient conversion of dietary nitrogen to microbial protein synthesis and improved growth performance in ruminants^10^. Although starch digestibility was not specifically measured in this study, it is likely that the higher digestibility of SFC-containing starter compared to GC may have contributed to the lower blood urea-N concentration observed in the treatment group (Table 3). This suggests improved utilization of nitrogen, although there was no difference in ammonia-N concentration between treatments.

The increased intake of starter and higher nutrient digestibility observed in calves receiving SFC-based starter compared with those receiving GC-based starter suggests that the former provided more metabolizable energy to support calf growth. The lower NDF digestibility in calves receiving GC-based starters compared to those receiving SFC-based starters may indicate incomplete rumen fermentation in the former group, as a metabolically functional rumen is necessary for efficient digestion of fibrous components in young calves^19^. The higher concentration of total VFA in the rumen as well as propionate and butyrate in calves fed SFC-based starters suggests that rumen fermentation was better developed in these calves after weaning. Both propionate and butyrate have been shown to induce rumen growth development^4^ and provide more energy per molar equivalent than acetate^64^, so they tend to improve growth performance^3^.

The lowest NDF digestibility was observed in calves fed GC75, suggesting that feeding GC-based starter diets in combination with late weaning may impair the development of a metabolically functional rumen and limit fiber digestion. The poorer feed efficiency observed in late-weaned calves compared with early-weaned calves may also indicate a less developed rumen that is less able to digest nutrients. These data suggest that extending weaning age beyond 50 days does not favor gastrointestinal tract maturation or increase capacity for nutrient digestion. However, these conclusions should be taken with caution because blood BHB and VFA concentrations, both indicators of calf rumen development, were not different between early and late weaned calves. Further measurements are needed to accurately determine the indicators of calf rumen development.

### Behavior

Consistent with our findings that SFC-containing starter feed increases the time calves spend eating, Rastgoo et al.^8^ reported that Holstein calves spend more time eating and tend to ruminate more in the preweaning and postweaning periods when fed SFC-containing starter feed compared with GC. This difference in feeding behavior was attributed to the larger particle size of SFC compared to GC. However, in our study, despite the difference in particle size of the calf starter feed, no differences in rumination behavior were observed between treatments. Further studies are needed to understand the relationship between calf feeding behavior and corn processing in forage-based starter feeds, as the type, amount, and particle size of the feed are also critical factors in calf rumination behavior. Consistent with this, Mojahedi et al.^16^ found no differences in the time calves spent eating when fed starter diets containing SFC or GC with or without alfalfa hay.

## Conclusions

Based on our findings, the type of corn processing had a greater effect on nutrient digestion than weaning age. Calves fed starter feed containing SFC consumed more starter feed and grew faster compared to GC, probably due to better digestibility of nutrients. Delayed weaning from 50 to 75 days resulted in calves growing faster and converting nutrients to growth more efficiently. Analysis of interaction effects between corn processing method and age at weaning indicated that calves at both early and late weaning benefited from inclusion of SFC in starter feed, as evidenced by greater starter intake, better nutrient digestion, improved growth performance, and lower blood urea-nitrogen concentrations, likely indicative of improved nitrogen utilization efficiency.

## Data Availability

The datasets used and/or analyzed during the current study are available from the corresponding author on reasonable request.

## References

[1] Khan MA, Bach A, Weary DM, von Keyserlingk MAG (2016). Invited review: Transitioning from milk to solid feed in dairy heifers. J. Dairy Sci..

[2] Porter JC, Warner RG, Kertz AF (2007). Effect of fiber level and physical form of starter on growth and development of dairy calves fed no forage. Prof. Anim. Sci..

[3] Makizadeh H (2020). Corn processing and crude protein content in calf starter: Effects on growth performance, ruminal fermentation, and blood metabolites. J. Dairy Sci..

[4] Baldwin RL, McLeod KR, Klotz JL, Heitmann RN (2004). Rumen development, intestinal growth and hepatic metabolism in the pre- and postweaning ruminant. J. Dairy Sci..

[5] Mirzaei M (2016). Interactions between the physical form of starter (mashed versus textured) and corn silage provision on performance, rumen fermentation, and structural growth of Holstein calves. J. Anim. Sci..

[6] Pazoki A (2017). Growth performance, nutrient digestibility, ruminal fermentation, and rumen development of calves during transition from liquid to solid feed: Effects of physical form of starter feed and forage provision. Anim. Feed Sci. Technol..

[7] Moeini H (2016). Effects of physical form of starter and forage provision to young calves on blood metabolites, liver composition and intestinal morphology. J. Anim. Physiol. Anim. Nutr..

[8] Rastgoo M, Kazemi-Bonchenari M, HosseinYazdi M, Mirzaei M (2020). Effects of corn grain processing method (ground versus steam-flaked) with rumen undegradable to degradable protein ratio on growth performance, ruminal fermentation, and microbial protein yield in Holstein dairy calves. Anim. Feed Sci. Technol..

[9] Offner A, Bach A, Sauvant D (2003). Quantitative review of in situ starch degradation in the rumen. Anim. Feed Sci. Technol..

[10] Zinn RA, Owens FN, Ware RA (2002). Flaking corn: Processing mechanics, quality standards, and impacts on energy availability and performance of feedlot cattle. J. Anim. Sci..

[11] Warner RG, Flatt WP, Loosli JK (1956). Ruminant nutrition, dietary factors influencing development of ruminant stomach. J. Agric. Food Chem..

[12] Sander E, Warner R, Harrison H, Loosli J (1959). The stimulatory effect of sodium butyrate and sodium propionate on the development of rumen mucosa in the young calf. J. Dairy Sci..

[13] Bateman HG, Hill TM, Aldrich JM, Schlotterbeck RL (2009). Effects of corn processing, particle size, and diet form on performance of calves in bedded pens. J. Dairy Sci..

[14] Ghassemi Nejad J (2012). Effects of processing of starter diets on performance, nutrient digestibility, rumen biochemical parameters and body measurements of brown swiss dairy calves. Asian-Australas. J. Anim. Sci..

[15] Beharka AA, Nagaraja TG, Morrill JL, Kennedy GA, Klemm RD (1998). Effects of form of the diet on anatomical, microbial, and fermentative development of the rumen of neonatal calves. J. Dairy Sci..

[16] Mojahedi S (2018). Performance, nutritional behavior, and metabolic responses of calves supplemented with forage depend on starch fermentability. J. Dairy Sci..

[17] NASEM. (National Academies of Sciences, E., and Medicine). *Nutrient Requirements of Dairy Cattle (8th rev. ed.)*. (National Academies Press, 2021). 38386771

[18] Roth BA, Keil NM, Gygax L, Hillmann E (2009). Influence of weaning method on health status and rumen development in dairy calves. J. Dairy Sci..

[19] Quigley JD, Hill TM, Dennis TS, Suarez-Mena FX, Schlotterbeck RL (2018). Effects of feeding milk replacer at 2 rates with pelleted, low-starch or texturized, high-starch starters on calf performance and digestion. J. Dairy Sci..

[20] Chapman CE (2016). Effect of milk replacer program on calf performance and digestion of nutrients with age of the dairy calf. J. Dairy Sci..

[21] de Passillé AM, Borderas TF, Rushen J (2011). Weaning age of calves fed a high milk allowance by automated feeders: Effects on feed, water, and energy intake, behavioral signs of hunger, and weight gains. J. Dairy Sci..

[22] Eckert E, Brown HE, Leslie KE, DeVries TJ, Steele MA (2015). Weaning age affects growth, feed intake, gastrointestinal development, and behavior in Holstein calves fed an elevated plane of nutrition during the preweaning stage. J. Dairy Sci..

[23] Meale SJ (2017). Weaning age influences the severity of gastrointestinal microbiome shifts in dairy calves. Sci. Rep..

[24] Rosenberger K, Costa JHC, Neave HW, von Keyserlingk MAG, Weary DM (2017). The effect of milk allowance on behavior and weight gains in dairy calves. J. Dairy Sci..

[25] Bjorklund EA, Heins BJ, Chester-Jones H (2013). Whole-milk feeding duration, calf growth, and profitability of group-fed calves in an organic production system. J. Dairy Sci..

[26] Lopreiato V (2020). Post-weaning rumen fermentation of Simmental calves in response to weaning age and relationship with rumination time measured by the Hr-Tag rumination-monitoring system. Livest. Sci..

[27] Jasper J, Weary DM (2002). Effects of ad libitum milk intake on dairy calves. J. Dairy Sci..

[28] Khan MA (2007). Structural growth, rumen development, and metabolic and immune responses of holstein male calves fed milk through step-down and conventional methods. J. Dairy Sci..

[29] Hill TM, Aldrich JM, Schlotterbeck RL, Bateman HG (2006). Effects of feeding calves different rates and protein concentrations of twenty percent fat milk replacers on growth during the neonatal period. Prof. Anim. Sci..

[30] Hu W (2020). Effects of milk replacer feeding rates on growth performance of Holstein dairy calves to 4 months of age, evaluated via a meta-analytical approach. J. Dairy Sci..

[31] Vitali A (2009). Seasonal pattern of mortality and relationships between mortality and temperature-humidity index in dairy cows. J. Dairy Sci..

[32] Bielmann V (2010). An evaluation of Brix refractometry instruments for measurement of colostrum quality in dairy cattle. J. Dairy Sci..

[33] Plascencia A, Zinn RA (1996). Influence of flake density on the feeding value of steam-processed corn in diets for lactating cows. J. Anim. Sci..

[34] AOAC. Official Method of Analysis. 16th Edition, Association of Official Analytical. Washington DC*.* (2002).

[35] Van Keulen J, Young BA (1977). Evaluation of acid-insoluble ash as a natural marker in ruminant digestibility studies. J. Anim. Sci..

[36] Van Soest PJ, Robertson JB, Lewis BA (1991). Methods for dietary fiber, neutral detergent fiber, and nonstarch polysaccharides in relation to animal nutrition. J. Dairy Sci..

[37] Zhu L (2016). An evaluation of total starch and starch gelatinization methodologies in pelleted animal feed. J. Anim. Sci..

[38] Khademi AR, Hashemzadeh F, Khorvash M (2022). Use of exogenous fibrolytic enzymes and probiotic in finely ground starters to improve calf performance. Sci. Rep..

[39] Lesmeister KE, Heinrichs AJ (2005). Effects of adding extra molasses to a texturized calf starter on rumen development, growth characteristics, and blood parameters in neonatal dairy calves. J. Dairy Sci..

[40] Bal MA, Shaver RD, Jirovec AG, Shinners KJ, Coors JG (2000). Crop processing and chop length of corn silage: Effects on intake, digestion, and milk production by dairy cows. J. Dairy Sci..

[41] Lesmeister KE, Heinrichs AJ (2004). Effects of corn processing on growth characteristics, rumen development, and rumen parameters in neonatal dairy calves. J. Dairy Sci..

[42] Ansari M (2022). Potential benefits of early-life supplementation of liquid feed with fennel (Foeniculum vulgare) seeds or oregano (Origanum vulgare) leaves on growth, health, and blood metabolites in Holstein dairy calves. J. Dairy Sci..

[43] Akbarian-Tefaghi M (2022). Effects of freestall vs. bedded pack housing on growth performance, health status, and behavioral responses of weaned heifers. Res. Vet. Sci..

[44] Miller-Cushon EK, DeVries TJ (2011). Effect of early feed type exposure on diet-selection behavior of dairy calves. J. Dairy Sci..

[45] Ghaffari MH, Kertz AF (2021). Review: Effects of different forms of calf starters on feed intake and growth rate: A systematic review and Bayesian meta-analysis of studies from 1938 to 2021. Appl. Anim. Sci..

[46] Omidi-Mirzaei H, Azarfar A, Kiani A, Mirzaei M, Ghaffari MH (2018). Interaction between the physical forms of starter and forage source on growth performance and blood metabolites of Holstein dairy calves. J. Dairy Sci..

[47] Imani M, Mirzaei M, Baghbanzadeh-Nobari B, Ghaffari MH (2017). Effects of forage provision to dairy calves on growth performance and rumen fermentation: A meta-analysis and meta-regression. J. Dairy Sci..

[48] Ahmadi F, Akbarian-Tefaghi M, Jafari A, Ghaffari MH (2022). Effects of different milk feeding levels and frequencies on performance of Holstein heifers during weaning and first lactation. Sci. Rep..

[49] Ahmadi F, Ghasemi E, Alikhani M, Akbarian-Tefaghi M, Hosseini Ghaffari M (2022). Effects of group housing and incremental hay supplementation in calf starters at different ages on growth performance, behavior, and health. Sci. Rep..

[50] Mirzaei M (2018). Effects of preweaning total plane of milk intake and weaning age on intake, growth performance, and blood metabolites of dairy calves. J. Dairy Sci..

[51] Palmquist D, Doppenberg J, Roehrig K, Kinsey D (1992). Glucose and insulin metabolism in ruminating and veal calves fed high and low fat diets. Domest. Anim. Endocrinol..

[52] Young J (1977). Gluconeogenesis in cattle: Significance and methodology. J. Dairy Sci..

[53] de Paula MR (2017). Intensive liquid feeding of dairy calves with a medium crude protein milk replacer: Effects on performance, rumen, and blood parameters. J. Dairy Sci..

[54] McCoard S (2019). Effect of weaning age on growth, mammary gland development, and immune function in Holstein Friesian calves fed conserved alfalfa (FiberStart). J. Dairy Sci..

[55] Greenwood RH, Morrill JL, Titgemeyer EC, Kennedy GA (1997). A new method of measuring diet abrasion and its effect on the development of the forestomach. J. Dairy Sci..

[56] Laarman AH, Oba M (2011). Short communication: Effect of calf starter on rumen pH of Holstein dairy calves at weaning. J. Dairy Sci..

[57] Aragona KM (2020). Effect of starter form, starch concentration, and amount of forage fed on Holstein calf growth from 2 to 4 months of age. J. Dairy Sci..

[58] Krause KM, Combs DK, Beauchemin KA (2002). Effects of forage particle size and grain fermentability in midlactation cows. II. Ruminal pH and chewing activity. J. Dairy Sci..

[59] Clark JH, Klusmeyer TH, Cameron MR (1992). Microbial protein synthesis and flows of nitrogen fractions to the duodenum of dairy cows. J. Dairy Sci..

[60] van Niekerk JK (2020). Effect of amount of milk replacer fed and the processing of corn in starter on growth performance, nutrient digestibility, and rumen and fecal fibrolytic bacteria of dairy calves. J. Dairy Sci..

[61] Ghorbani H, Kazemi-Bonchenari M, HosseinYazdi M, Mahjoubi E (2020). Effects of various fat delivery methods in starter diet on growth performance, nutrients digestibility and blood metabolites of Holstein dairy calves. Anim. Feed Sci. Technol..

[62] Quigley JD (2019). Symposium review: Re-evaluation of National Research Council energy estimates in calf starters. J. Dairy Sci..

[63] Corona L, Rodriguez S, Ware RA, Zinn RA (2005). Comparative effects of whole, ground, dry-rolled, and steam-flaked corn on digestion and growth performance in feedlot cattle. Prof. Anim. Sci..

[64] Bergman E (1990). Energy contributions of volatile fatty acids from the gastrointestinal tract in various species. Physiol. Rev..

